# Blocking long noncoding RNA MALAT1 restrained the development of laryngeal and hypopharyngeal carcinoma

**DOI:** 10.1007/s00405-019-05732-x

**Published:** 2019-12-02

**Authors:** Enhong Xu, Xiaoben Liang, Zhenhua Ji, Shuwei Zhao, Li Li, Juntian Lang

**Affiliations:** 1 Department of Otolaryngology Head and Neck Surgery, Changzheng Hospital, Second Military Medical University, Shanghai, China; 2 Department of Otolaryngology Head and Neck Surgery, Children’s Hospital of Shanghai, Shanghai, China

**Keywords:** Long non-coding RNA, Laryngeal squamous cell carcinoma, Hep-2 cell, FaDu cell, MicroRNA

## Abstract

**Purpose:**

The long non-coding RNA MALAT1 is a predictive marker in several solid tumors with highly conserved sequences. However, the role of non-coding RNA in development of laryngeal or hypopharyngeal cancer remains unclear.

**Methods:**

Tumor tissues and adjacent non-cancer tissues of 24 patients were collected. We detected the expression of MALAT1 in laryngeal cancer tissues and hypopharyngeal cancer tissues. Moreover, we developed a MALAT1 silencing model in human laryngeal tumor cells by transfecting MALAT1 small interfering RNA into human laryngeal carcinoma cell line Hep-2 and pharyngeal carcinoma cell line FaDu with Lipofectamine 2000 system. Cell cycle analysis, Cell Counting Kit-8 assay, Transwell assay, quantitative reverse transcription PCR, and wound-healing assays were performed to evaluate the impact of MALAT1 depletion on laryngeal or hypopharyngeal cancer cell’s growth, proliferation, apoptosis, invasion and migration.

**Results:**

MALAT1 was significantly up-regulated in laryngeal and hypopharyngeal carcinoma cells. MALAT1 down-regulation induced the increased apoptosis of both cell lines and suppressed cells’ proliferation. Cells were arrested in G1/G2 phase and cells of S phase were significantly decreased. Down-regulation of MALAT1 expression can also inhibit the migration and invasion of laryngeal squamous cell carcinoma cell (Hep-2) and hypopharyngeal cancer cell (FaDu).

**Conclusion:**

In summary, our deactivation model of MALAT1 disentangled the active function of it as a regulator of gene expression governing the hallmarks of laryngeal and hypopharyngeal cancer. Blocking this long non-coding RNA may restrain the development of laryngeal cancer.

## Introduction

Laryngeal squamous cell carcinoma (LSCC) is one of the most common malignancies of head and neck, accounting for 7.9–35% of the otolaryngology tumors [1]. In recent years, the incidence of LSCC increased remarkably. Early laryngeal cancer can be cured by a variety of treatments, while advanced laryngeal cancer has poor prognosis with high mortality. The development mechanism of LSCC remains unknown, because multiple genes and functional RNA may participate in this complicated molecular regulation pathway. Recently, the role of non-coding RNA in development of human cancer has been revealed.

The long non-coding RNAs (lncRNAs) are defined by length, ranging from 200 nt to 138 kb. Like messenger RNAs, they are transcribed by RNA polymerase II and can be modified by diverse ways. Nevertheless, lncRNAs lack a significant open reading frame, which can be transcribed from the sense or antisense orientation [2]. LncRNAs are important molecular elements in eukaryotic cells, and play key roles in various aspects of mRNA stability, transcriptional regulation, protein transport, RNA processing and modification. Piling up evidence indicates that lncRNAs play a critical role in multiple cancers development and progression. Lung adenocarcinoma metastasis-associated transcript 1 (MALAT1) is one of the earliest discovered lncRNAs, which is about 8700 bp, and located in human 11q13 chromosome. It was originally screened by subtractive hybridization in a study of non-small cell lung cancer (NSCLC) [3]. MALAT1 has evolutionary conservation in its sequence and highly homologous sequences among species, suggesting that it has an important function in tumorigenesis and development [4]. Although MALAT1 has been investigated in multiple human cancers [5–7], it is rarely known whether it is associated with laryngeal cancer development in some mechanisms. In this study, we detect the expression of MALAT1 in laryngeal squamous cell carcinoma and hypopharyngeal cancer. In addition, we probed the function of MALAT1 in the development of laryngeal and hypopharyngeal cancer by developing a MALAT1 silencing model in human laryngeal tumor cells.

## Materials and methods

### Tissue specimens and clinical data

Following the Declaration of Helsinki for medical ethics, the present study was under the approval of the ethics committee of Changzheng Hospital, the Second Military Medical University. Written informed consent was obtained from all patients involved. Twenty-four primary laryngeal and hypopharyngeal squamous cell carcinoma (LHSCC) specimens surgically resected at the Department of Otolaryngology Head and Neck Surgery in Changzheng Hospital (Shanghai, China) from 2013 to 2016 were analyzed. Patients included 21 males and 3 females with a mean age of 58.3 years (ranged from 40 to 75 years). No patients underwent radiation or chemotherapy before surgery. A total of 48 tissue samples (24 LHSCC tumor tissue samples and paired adjacent healthy tissue samples) were obtained during surgery. Adjacent healthy tissue was defined as mucosa tissues if it was at least 3 cm far from the edge of tumor. All resected tissue samples were divided into two parts, one was preserved in liquid nitrogen (− 180 °C) within 5 min from resection and the other part was fixed in 10% formalin and embedded in paraffin according to routine laboratory protocols. Meanwhile, the para-cancerous tissues more than 2 cm from the edge of the cancer tissue were collected as control. Baseline characteristics were reasonably balanced between treatment aims and are presented in Table 1.

**Table 1:** Baseline characteristics of the patient population

Parameter	Patients (*N* = 24)	Laryngeal carcinoma (*N* = 16)	Hypopharynx carcinoma (*N* = 8)
Age (years)	Median	59	57
	Range	40–75	42–68
Gender, *N* (%)	*n*	16	8
	Male	15 (93.75)	6 (75)
	Female	1 (6.25)	2 (25)
Stage (TNM^a^), *N* (%)	*n*	16	8
	I	3 (18.75)	1 (12.5)
	II	3 (18.75)	1 (12.5)
	III	4 (25)	1 (12.5)
	IV	6 (37.5)	5 (62.5)
Primary tumor site, *N* (%)	*n*	16	8
	Supraglottis	4 (25)	
	Glottis	10 (62.5)	
	Subglottic	2 (12.5)	
	Piriform sinus		6 (75)
	Postericoid region		1 (12.5)
	Posterior hypopharynx		1 (12.5)

^a^TNM clinical staging was based on National Comprehensive Cancer Network Guidelines Version 1.2019

### RNA isolation

Total RNA of tissue samples was extracted using RNA Isolation Kit (RP4001, BioTeke, Beijing, China) according to the manufacturer’s recommendation, The RNA quantity and purity were determined using the NanoDrop 1000 Spectrophotometer (Thermo Scientific, Wilmington, DE, USA), with a sample of 1.5 μl. The RNA specimens were finally dissolved in 40 μl of nuclease-free water, stored at − 80 °C until use.

### Quantitative reverse transcription-polymerase chain reaction

Quantitative reverse transcription-polymerase chain reaction (qRT-PCR) kits were used to evaluate the expression levels of the selected lncRNA. PCR was performed as previously described [8]. The primers used in this study are shown below: MALAT1 forward 5′-CTGGAGAAGATAGGCATT-3′; reverse 5′-CCAAGTCTGTTATGTTCAC-3′.

β-actin forward 5′-CATGTACGTTGCTATCCAGGC-3′; reverse 5′-CTCCTTAATGTCACGCACGAT-3.

All of the reactions were performed with the following conditions: 94 °C for 30 s, followed by 40 cycles of 94 °C for 10 s, 60 °C for 30 s. Samples were analyzed in triplicate and included no-template controls. Amplification of the appropriate product was validated by a melting curve analysis. The relative expression of MALAT1 was calculated using the comparative cycle threshold (CT, 2^−ΔΔCT^) method.

### RNA interference

Cells were divided into two groups, one was transfected with siRNAs targeting MALAT1, while the other group was control. Lipofectamine 2000 (Invitrogen, Carlsbad, CA, US) was employed to perform the RNA interference following manufacturer’s instructions. For the intronic MALAT1 knockdown, a pool of ten siRNAs (sense 5′-3′: CCCUCUAAAUAAGGAAUATT, antisense 3′-5′: UUAUUCCUUAUUUAGAGGGTT, labeled with 5′FAM) targeting intronic regions in MALTA1 were transfected into cells, and RNA isolated as above. 100 pmol NC (normal control) group, 100 pmol si-MALAT1 and 5 µl Lipofectamine 2000 were diluted by 250 µl Opti-MEM, then 100 pmol NC group and 5 µl Lipofectamine 2000 were mixed and finally 100 pmol si-MALAT1 group and 5 µl Lipofectamine 2000 mixed. Twenty minutes after incubation at room temperature, the above compounds were mixed with cells (NC group/si-MALAT1 group), and cultivated at 37 °C, 5% CO_2_ in DMEM + 10% FBS. qRT-PCR was performed (following the method described above) to determine the effect of MALAT1 interference in both cell groups.

### Cell culture and cell cycle analysis

Hep-2 cells and FaDu cells were obtained from Shanghai Changhai Hospital and cultivated at 37 °C, 5% CO_2_ in DMEM + 10% fetal bovine serum (FBS). Cells were plated onto a 6-well plate at a density of 5 × 10^5^ cells/well and grown for 24 h. The cells were then starved with serum-free culture medium for 24 h to synchronize them at the G1/S boundary, followed by transfection. After 48 h, the cells were collected, washed twice with ice-cold PBS, and fixed in 70% ice-cold ethanol at 4 °C overnight. After rehydration in PBS for 15 min, the cells were stained for 30 min in the dark with propidium iodide solution. Further, cells were stained with Annexin-V FITC and propidium iodide, according to the manufacturer’s instructions. The apoptosis rate was analyzed by flow cytometry (BD Biosciences, New York, USA). The experiments were independently performed in triplicate.

### Cell proliferation assay

Cells were harvested at the logarithmic growth phase and used to prepare single cell suspensions. Cells were dyed with crystal violet (Sigma-Aldrich; Merck KGaA, Germany). Cell density was adjusted and cells were transferred into 96-well plates at a density of 4 × 10^3 ^cells/well. Cells were cultured under normal conditions (37 °C, 5% CO_2_), and 10 μl of Cell Counting Kit-8 (CCK-8) solution (Sigma-Aldrich, Merck KGaA, Germany) was added in proper sequence at 24, 48, 72 and 96 h later. Five hours later, incubation was performed and then the optical density values (450 nm) were measured using a microplate reader.

### Transwell cell migration and invasion assay

Invasion ability was measured with 24-well Transwell chamber as previous described [9]. Cells were digested, cleaned with DMEM and suspended to 1 × 10^5^ cells/ml. Then, 200 μl of 5 × 10^5^ cells were seeded in the upper chamber, while 600 μl of 50 ug/ml Fibronectin (Corning, USA) supplemented with 10% FBS was used to fill the lower chamber. Cells were incubated under normal conditions, before the membranes were cleaned with PBS and dyed with crystal violet (Sigma-Aldrich; Merck KGaA, Germany). Dyed cells were counted under an optical microscope.

### Wound-healing assay

Cells were seeded in 6-well plates at an initial density of 2 × 10^5^ cells/well and grown to about 80% confluence [9]. A vertical wound was created by scratching the monolayer with a sterile 200-μl pipette tip, and the cells were then washed with PBS three times. The monolayer was subsequently incubated in serum-free medium. The area of the scratched surface between the edges of defect was measured immediately after disruption and after 24, 48 and 72 h. Photographs were taken with a microscope at 200× magnification (Nikon, Japan) at the same location in each well to monitor cell migration into the wounded area. Wound area was calculated by manually tracing the cell-free area in captured images using the ImageJ public domain software (NIH, Bethesda, MD) [10]. The experiments were independently performed in triplicate.

### Statistical analysis

Statistical analyses were performed using SPSS 25.0 (IBM, Chicago, USA) and data are presented as the mean ± standard deviation. An unpaired two-tailed Student’s *t* test or one-way ANOVA (analysis of variance) with Bonferroni’s post-test were used to analyze the data as appropriate. *P* < 0.05 was considered to indicate a statistically significant difference.

## Results

### MALAT1 was up-regulated in laryngeal and hypopharyngeal cancer tissues

**Fig. 1:**
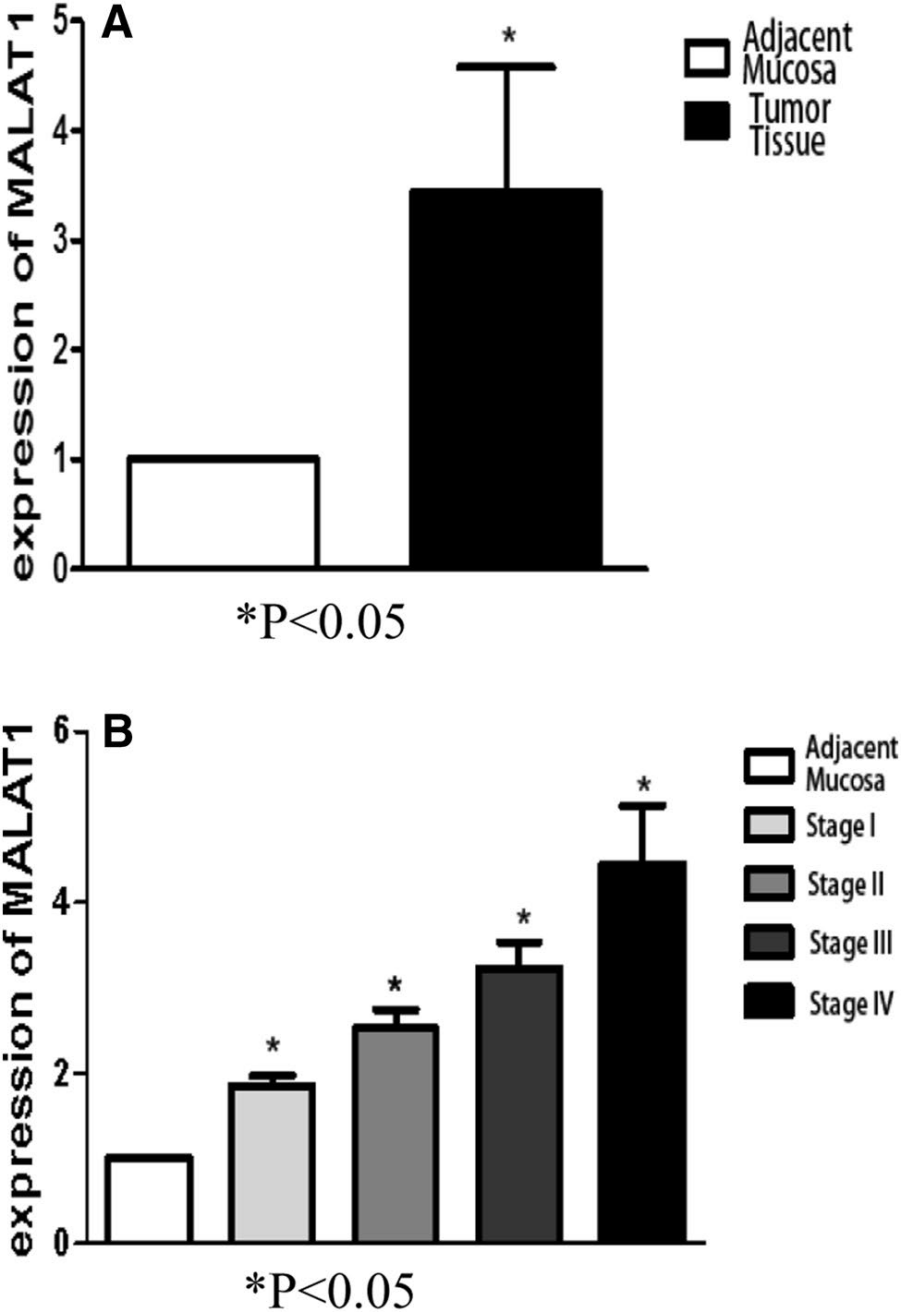
MALAT1 expression in tumor and non-tumor tissues was measured by qRT-PCR. **a** MALAT1 expression was significantly higher in laryngeal cancer and hypopharyngeal cancer tissues compared with that in the healthy tissues. **b** The expression of MALAT1 among different stages of tumor was significantly different, with a high expression of MALAT1 frequently occurring in LHSCC at an advanced stage

To investigate the role of MALAT1 in LHSCC, we collected microarrays consisting of 24 fresh laryngeal/pharyngeal cancer tissue samples and corresponding para-cancer normal tissue samples. The total RNA of each specimen was extracted. Further, the gene expression levels were detected by real-time PCR. The ISH data illustrated that MALAT1 expression was significantly increased in laryngeal cancer and hypopharyngeal tissues as compared with that in the normal tissues (*P* < 0.05, Fig. 1). Furthermore, the expression of MALAT1 at different cancer stages was investigated. Consequently, it was found that higher MALAT1 expression level frequently occurs in LHSCC at an advanced stage (*P* < 0.05), indicating an expectancy that MALAT1 may act as an oncogene in the development of LHSCC and may be a biomarker indicating tumor advance.

### RNA interference of MALAT1 effectively suppresses the level of MALAT1 in Hep-2 and FaDu cell lines

**Fig. 2:**
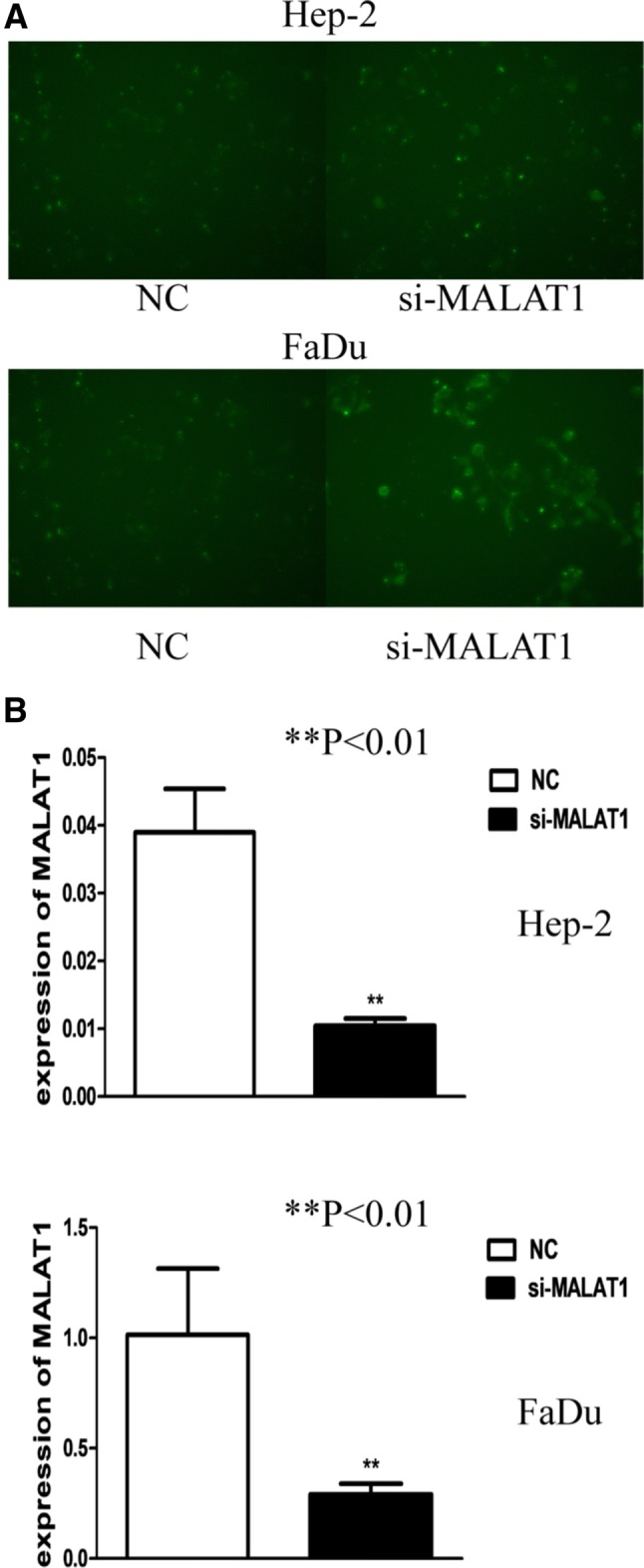
MALAT1 was down-regulated after si-MALAT1 interference. **a** Fluorescence microscope photos (200× ) show that 5′FAM-labeled siRNA was observed in si-MALAT1 transfected cell group, 48 h after interference. **b** Quantitative RT-PCR detection showed that the expression of MALAT1, 48 h after si-MALT1 interference, was down-regulated by 73.2% compared to the NC group(*P* < 0.01). Also, down-regulation was observed in FaDu cells (by 71.3%, *P* < 0.01)

To explore the relationship between MALAT1 and malignant biological properties of LHSCC in vitro, we knockdown the MALAT1 by RNA interference. Si-MALAT1 with fluorescence was transfected into Hep-2 cells and blank control group (NC group) was set up. After 48 h, fluorescence microscopy showed strong and dense green fluorescence. RT-PCR detection showed that the expression of MALAT1 after si-MALAT1 interference was down-regulated by about 73.2% (*P* < 0.01) compared with the NC group. In addition, we also observed a negative expression of MALAT1 (71.3% *P* < 0.01) in FaDu cells after interference compared with the control group (Fig. 2). The above result showed the RNA interference with Lipofectamine 2000 system was effectively down-regulating the MALAT1 expression.

### Si-MALAT1 inhibited the proliferation and induced the apoptosis of Hep-2 and FaDu cell lines

**Fig. 3:**
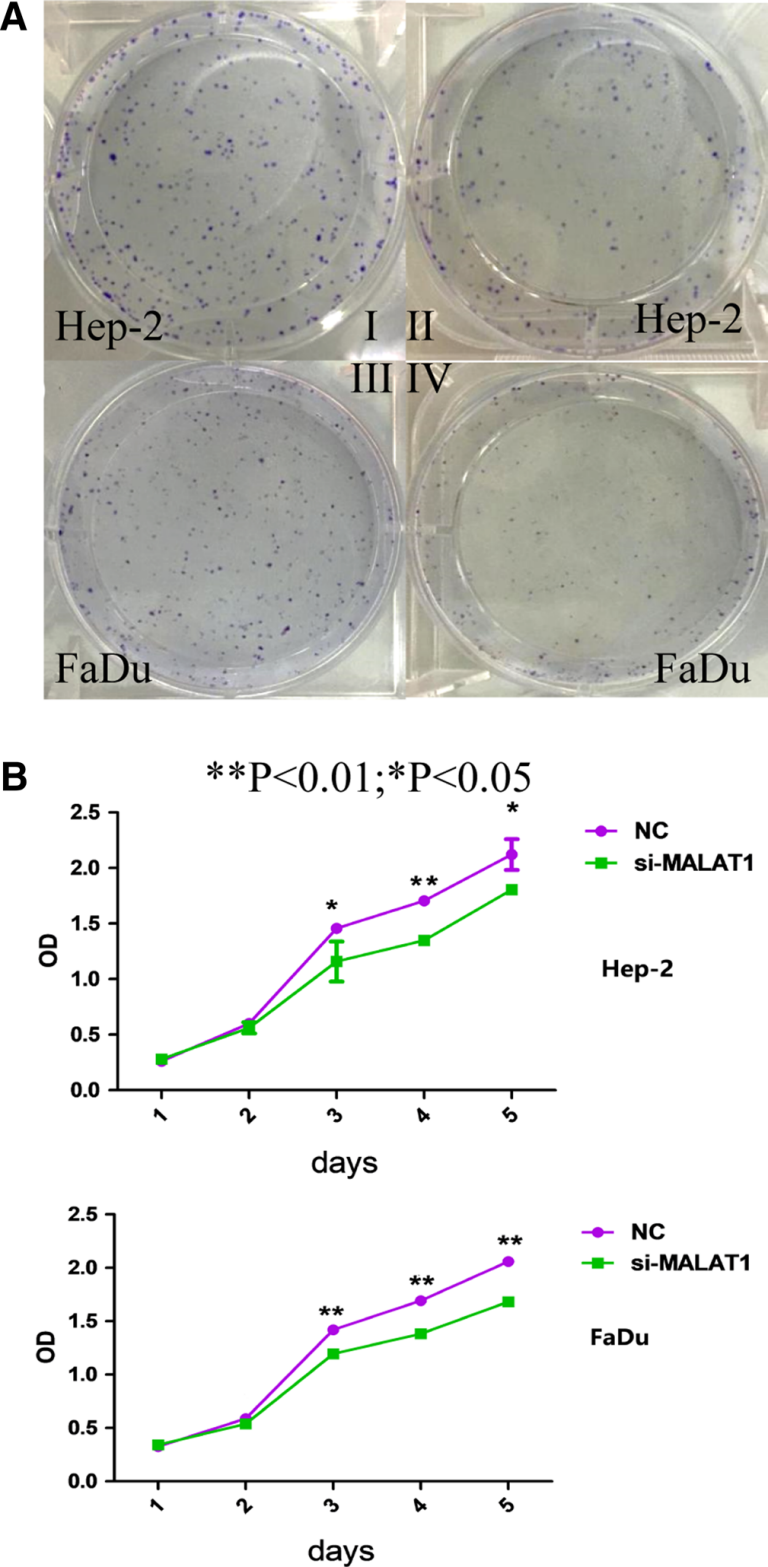
Cell proliferation was suppressed by si-MALAT1 interference. **a** Crystal violet staining shows significant difference of cells’ proliferation between si-MALAT1 (II/IV) group and NC group(I/III), 5 days after interference. **b** The results from the CCK-8 assays showed decreasing cell proliferation rate in both Hep-2 and FaDu cell lines. Significant difference was observed 3 days after interference (*P* < 0.01 for FaDu cell, and *P* < 0.05 for Hep-2)

**Fig. 4:**
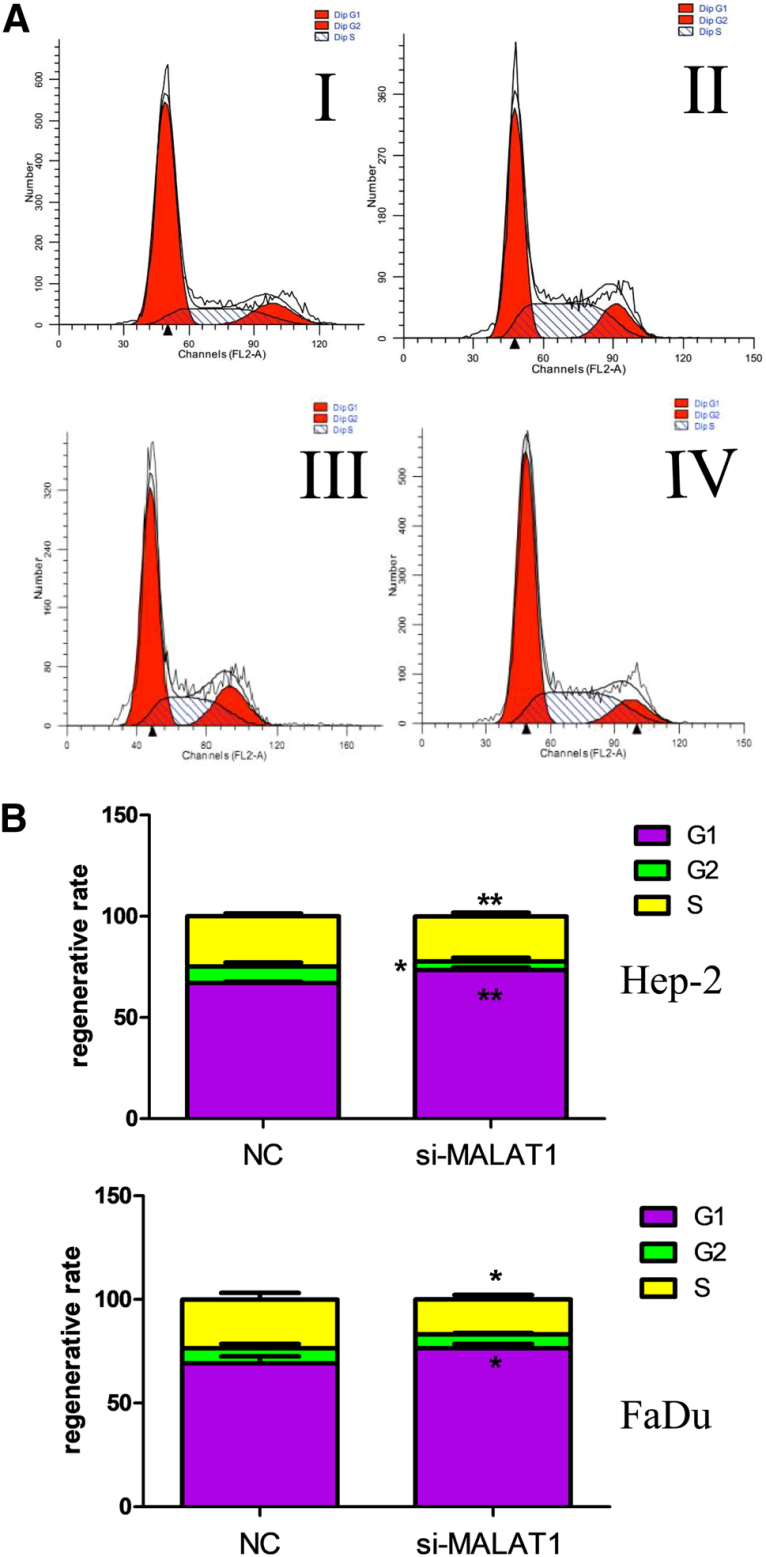
MALAT1 depletion led to cell cycle suspended in both Hep-2 and FaDu cell lines. **a** Flow cytometric analysis shows cell cycle was arrested at G1/G2 phase after si-MALAT1 interference (Hep-2 in I/II, FaDu in III/IV). **b** For both cell lines, after si-MALAT1 interference, much less cells entered into S phase (*P* < 0.05)

**Fig. 5:**
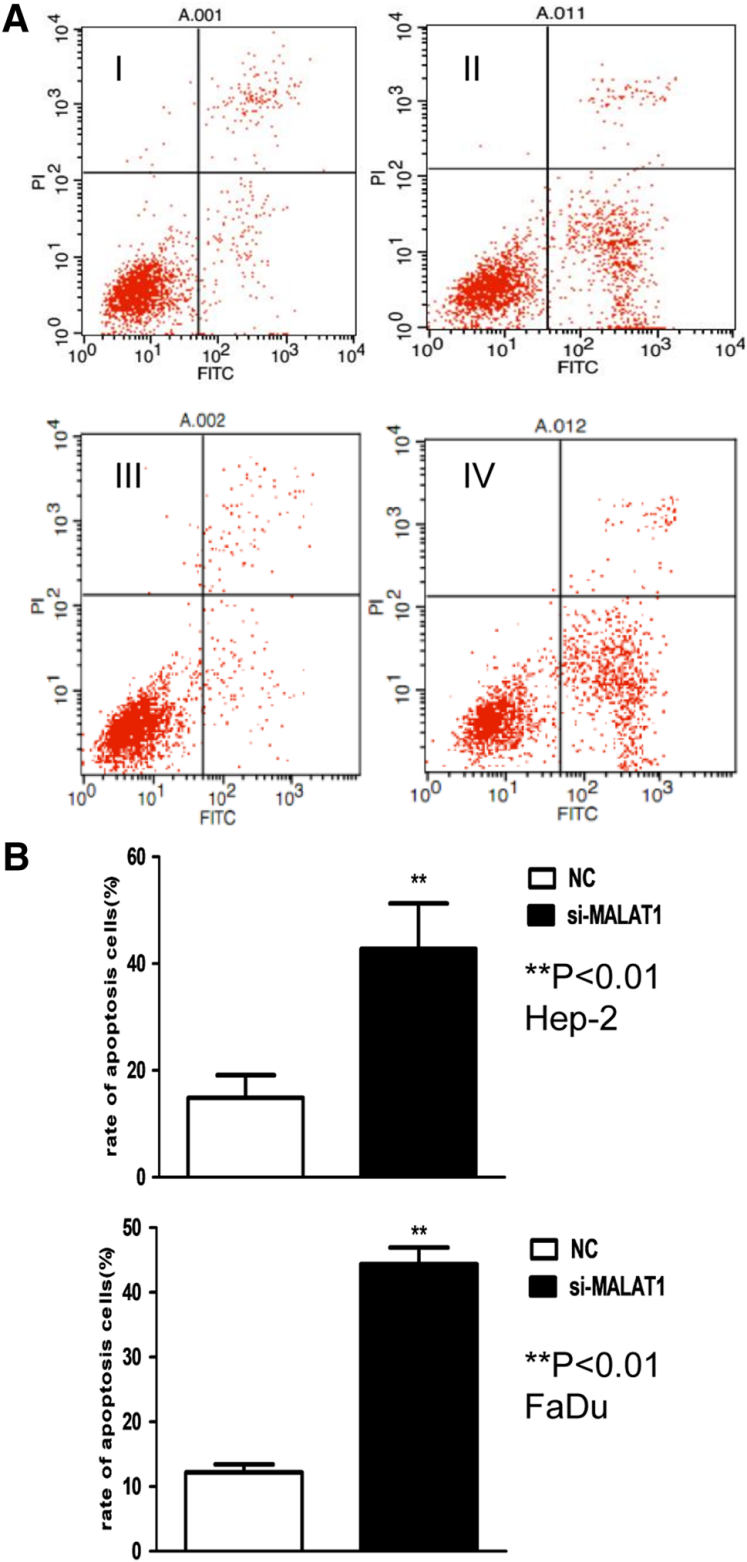
MALAT1 interference-induced cancer cell apoptosis increased. **a** Cell apoptosis was investigated by flow cytometric analysis (I—Hep-2 cell control group/II—Hep-2 cell si-MALAT1 group, III—FaDu cell control group/IV—FaDu cell si-MALAT1 group). **b** Both Hep-2 and FaDu cell lines apoptosis were significantly increased after si-MALAT1 interference (*P* < 0.01)

The impact of MALAT1 on the proliferation of Hep-2 and FaDu cells was investigated by MALAT1 RNA interference. Flow cytometric analysis showed that, after interference, suppression of MALAT1 expression accounted for dramatically inhibited cell proliferation as compared with that in the control (Fig. 3). In addition, flow cytometric analysis showed that MALAT1 depletion resulted in cell cycle arrested at G1/G2 phase in both Hep-2 and FaDu cell lines (Fig. 4). Moreover, for Hep-2 cell lines, much less cells entered into S phase after si-MALAT1 interference. Moreover, MALAT1 depletion also induced apoptosis in Hep-2 and FaDu cell lines (Fig. 5). Cell apoptosis was investigated by flow cytometric analysis. Both Hep-2 and FaDu cell lines apoptosis were significantly increased after si-MALAT1 interference.

### Si-MALAT1 suppressed the invasion and migration of Hep-2 and FaDu cell lines

**Fig. 6:**
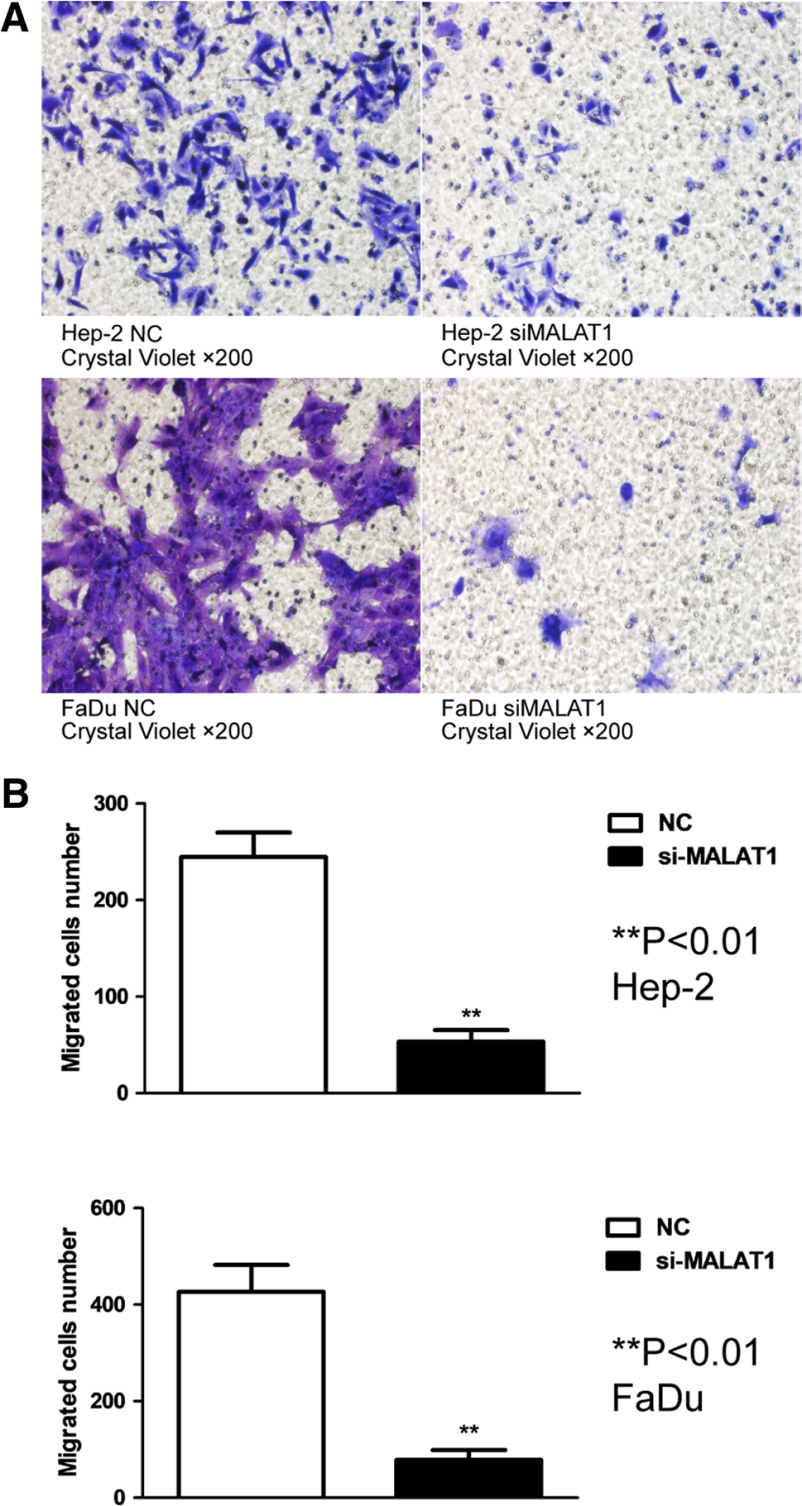
Silencing expression of MALAT1 impeded the invasion and migration of both Hep-2 and FaDu cell lines. **a** The Transwell chamber assay shows that there was less blue positive substance in the si-MALAT1 groups rather than that in control group (NC group). **b** Migrated cell number was evidently declined in study groups compared to NC groups, and the ratio (si-MALAT1/NC) was 0.22 in the Hep-2 group, and 0.18 in the FaDu group

**Fig. 7:**
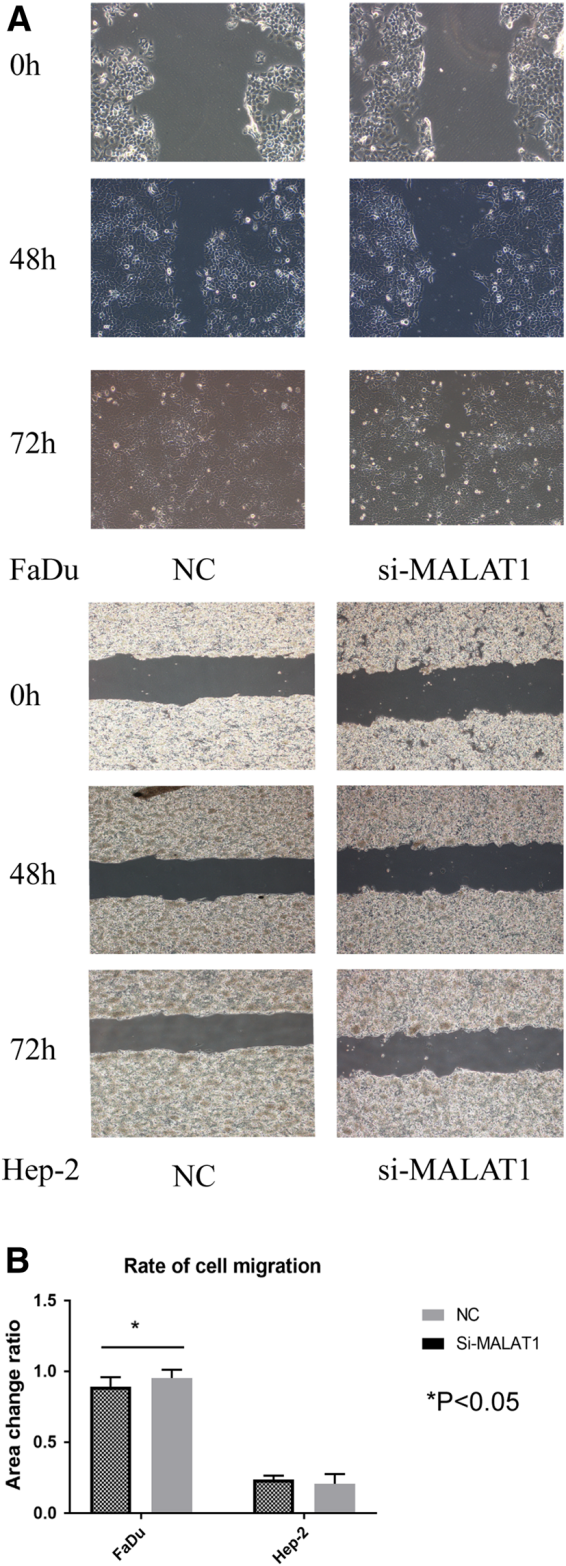
Down-regulating MALAT1 inhibited the migration of FaDu cells, but not significantly for Hep-2 cell. **a** Photos of scratching experiment. **b** The wound-healing assays demonstrated that area change ratios (calculated by scratching areas at 0 and 72 h) were significantly different between si-MALAT1 cells and controls for FaDu cell line (*P* < 0.05), but not for Hep-2 cell line (*P* > 0.05)

Transwell experiments and scratch experiments were performed to investigate whether lncRNA MALAT1 was involved in the invasion and migration of Hep-2 and FaDu cells. Indeed, we set three groups for each cell line. The result showed that silencing the expression of MALAT1 impeded invasion and migration both in Hep-2 and FaDu cell lines (Fig. 6). Migrated cell number was evidently declined in study groups compared to NC groups and the ratio (si-MALAT1/NC) was 0.22 in the Hep-2 group and 0.18 in the FaDu group. A wound-healing assay also demonstrated that down-regulating MALAT1 expression inhibited migration of FaDu cells (*P* < 0.05, Fig. 7), while not significantly for Hep-2 cell (*P* > 0.05).

## Discussion

MALAT1 was one of the primitively discovered cancer-associated lncRNA, also referred to as NEAT2 (Nuclear-Enriched Abundant Transcript 2). It was originally reported being associated with the development of NSCLC. Further, it was identified as a prognostic biomarker of NSCLC, specifically in early stages, for metastasis and patient survival [4, 11–13].

Numerous studies have uncovered aberrant expression of MALAT1 in a group of human tumor tissues, suggesting its significant role in many vital biological behavior including tumor cell proliferation, apoptosis, invasion and metastasis [4, 14–16]. These studies also pointed out that MALAT1 is up-regulated in oral cavity cancer, nasopharyngeal cancer and esophageal squamous cancer [17–19]. Although MALAT1 has been investigated in multiple human cancers, it is rarely known whether it is associated with laryngeal cancer development in some mechanisms.

In this study, we found that MALAT1 was significantly up-regulated in LHSCC tissues compared with normal tissues. Our data also show a hint that the expression level of MALAT1 may be linked to clinical stages of laryngeal cancer. Although due to the limited number of cases in this study, the association between MALAT1 expression and clinical staging of laryngeal cancer could not be confirmed, our result still shows the trend that the more advanced the stage, the higher the MALAT1 expression level will be. Our study is a pilot study, which is also not sufficient to elucidate the complicated role of MALAT1 in tumorigenesis and progression of laryngeal cancer. Further study with a large sample and multiple centers need to be carried out to identify this issue clearly. Not only cancer progression, but also tumor size, lymph node metastasis, and shorter overall survival of cancer patients were found to be associated with MALAT1 up-regulation [20–22]. Our results were in accordance with these studies. For example, Shen [21] reported that in non-small cell lung cancer, lncRNA GHET1 is associated with the survival of patients. Moreover, it might predict poor progression of patients with lung cancer. Their study showed that the overall survival (OS) in months of lncRNA GHET1 low-expression group is higher than the high-expression group. The progression-free survival (PFS) of patients with lncRNA GHET1 overexpression is shorter than that of the patients with low expression. Our findings imply that MALAT1 may be employed as a clinical prognostic biomarker for head and neck cancer progression.

As an important lncRNA, MALAT1 has been described as a decisive gene in various cancers regulating metastasis [3, 6, 23–27]. Qing et al. [28] found that MALAT1 had a significantly higher expression in recurrent colorectal cancer primary and metastatic tumors. Moreover, colorectal cancer patients with a higher level of MALAT1 in primary tumors had poor prognosis. Nevertheless, in the study by Kim et al., the authors showed that targeted inactivation of MALAT1 in a mouse model of breast cancer promoted lung metastasis [29], without altering the expression of its adjacent genes as previous researches did. Taken together, these studies imply the MALAT1 is associated with cancer progression in various ways.

We postulated that organs differ in the mechanism of MALAT1 involving the development and advance of cancer. In this study, the impact of MALAT-1 on laryngeal and hypopharyngeal cancer cell growth and proliferation was confirmed by RNA interference. Our results demonstrated that down-regulating MALAT1 can induce increased laryngeal cancer cell apoptosis and inhibition of cell proliferation. Hep-2/FaDu cells were arrested in G1/G2 phase and cells of S phase were significantly decreased. Down-regulation of MALAT1 expression decreased the invasion and migration of Hep-2/FaDu cells. A previous study has confirmed that MALAT1 might alter growth and colony formation of cancer cells in vitro [30]. Down-regulated MALAT1 impaired cell motility in vitro [31]. Upon injection into nude mice, cells with moderately decreased MALAT1 expression showed preclusion of tumors’ growth. The up-regulation of MALAT1 contributes to the proliferation and metastasis in esophageal squamous cell carcinoma [18]. In gastric cancer, MALAT1 could drive the development of cancer and promote peritoneal metastasis [32, 33]. In our study when depleting MALAT1 in laryngeal cancer cell lines, the invasion and migration of both laryngeal cancer cell lines Hep-2 and hypopharyngeal cancer cell FaDu were inhibited. Nonetheless, in a wound-healing assay, we noticed that silencing MALAT1 did not lead to significant diversity for Hep-2 cell lines. Thus, heterogeneity in these two cell lines should also be considered. And we will do further research to interpret this molecular mechanism. And more patients will be observed to unravel the clinical significance of MALAT1 in laryngeal and hypopharyngeal carcinoma.

The molecular mechanism of MALAT1 action is currently under debate [5–7].Previous studies suggested an intimate bond between MALAT1 and β-catenin signaling, but the detailed mechanism remains elusive [34].

Previous studies identified MALAT1 as a regulator of alternative splicing of gene subsets, while others proposed it as a mechanism of gene regulation [35]. According to Gutschner et al. [30], MALAT1 has no significant effect on alternative splicing of lung cancer cells, matching the latest data in mouse models [36, 37].

MALAT1 might regulate alternative splicing of a subset of pre-mRNAs by modulating serine / arginine splicing factor activity, which regulates tissue- or cell-type specific alternative splicing in a phosphorylation-dependent manner [38]. However, splicing alterations were not found after MALAT1 ablation in mice [37]. In contrast, alternative functions for MALAT1 were recently identified: MALAT1 could interact with the demethylated form of Chromobox homolog 4 (CBX4), also referred to as Polycomb 2 (Pc2), a component of the Polycomb Repressive Complex 1 (PRC1) [35, 39, 40]. This interaction controls the re-localization of growth control genes. MALAT1 resides in these subnuclear structures and acts as an activator of gene expression potentially by mediating the assembly of coactivator complexes [20]. In this way, MALAT1 influences proliferation, invasion and migration of cancer cells through regulation of multitudinous known downstream genes, including several metastasis-related genes (CCT4/CTHRC1/ROBO1/MIA2) and cell cycle control genes (p21/p27/B-MYB) [16].

MALAT1 was found to have a function in the development of cancer correlated with miRNAs. Meng Zhuang et al. demonstrated that MALAT1 might be a potential ceRNA by sponging miR-106b-5p in colorectal cancer. Accordingly, it promotes invasion and metastasis by regulating miR-106b-5p, which is in agreement with other authors’ findings [20, 32, 41, 42]. In hepatocellular carcinoma, MALAT1 is up-regulated and acts as a proto-oncogene via the activation of the Wnt pathway and induction of the oncogenic splicing factor SRSF [7]. However, these findings were challenged by the study of Han et al. [43], which showed that MALAT1 had a function as a tumor suppressor by attenuating the ERK/MAPK-mediated growth and MMP2-mediated invasiveness in glioma. Thus, in different tumors and different organs, MALAT1 may exert different roles in the tumorigenesis. The specific lncRNA was valuable in predicting the prognosis of colorectal cancer by investigating the correlations between MALAT1 and miR-106b-5p [22].

Taken together, we identified lncRNA MALAT1 as a novel prognostic biomarker for laryngeal and hypopharyngeal cancer. We first noticed the relationship between the expression of MALAT1 and stage of LHSCC. Our findings highlight the critical role of lncRNA MALAT1 for tumorigenesis and progression of head neck cancer cells. The mechanisms of laryngeal and hypopharyngeal squamous cell carcinoma development and progression are complicated and still unclear. Multiple genes and functional RNA may participate in this complicated molecular regulation pathway. Although our result shows that MALAT1 may act as a regulator of gene expression governing hallmarks of laryngeal and hypopharyngeal cancer, it is only one component in this comprehensive process and may carry out its role by working in coordination with other molecules and through a complex network. Additionally, it remains to be elucidated whether the ubiquitously expressed MALAT1 has one universal function or whether its mechanisms of action might be specifically different according to the tissues.
